# AGING IN THE RIGHT PLACE: TEMPORARY SUPPORTIVE HOUSING FOR OLDER MALE VETERANS IN CALGARY, CANADA

**DOI:** 10.1093/geroni/igad104.0208

**Published:** 2023-12-21

**Authors:** Jill Hoselton, Alison Grittner, Christine Walsh

**Affiliations:** University of Calgary, Calgary, Alberta, Canada; Cape Breton University, Sydney, Nova Scotia, Canada; University of Calgary, Calgary, Alberta, Canada

## Abstract

In 2019 an estimated 1.6% of emergency shelter users in Canada were veterans (1,905 individuals), which is consistent with the proportion of veterans in the general population (1.7%). However, veterans tend to be older, male, and cite an illness or medical condition as a contributing factor to their homelessness, than their non-veteran counterparts. As part of the Aging in the Right Place (AIRP) study, we sought to understand the housing and support needs of older male military veterans (age 50+) living in a congregate temporary supportive housing in Calgary, Alberta. This exploratory, multi-methods study used: (1) de-identified document review, (2) environmental audit, (3) in-depth qualitative key informant interviews with service providers and (4) in-depth qualitative and Photovoice interviews with older shelter residents to understand the shelter needs of older homeless veterans. The data was collected between February and June 2023. Interviews were transcribed, managed with NVivo 1.6.1, and team-based flexible coding was employed on the key informant (n=5) and shelter resident (n=5) interviews to determine older male veterans’ shelter needs. In this presentation we share the findings of the study and offer recommendations for shelter and services designed to assist older housing insecure veterans to age in the right place.

